# Chronic Edema Management of the Lower Extremities

**DOI:** 10.7759/cureus.63840

**Published:** 2024-07-04

**Authors:** Blake Smith, Jooheon Park, Jessica L Landi, Brandon McConnell, Akif Rahman, Abdul Rahman Omari, Zaynah Shahab, Andrew Carilli, Kaitlyn Pearl, Brian Kim, Joshua M Costin

**Affiliations:** 1 Medical School, Nova Southeastern University Dr. Kiran C. Patel College of Osteopathic Medicine, Fort Lauderdale, USA; 2 Medical Education, Nova Southeastern University Dr. Kiran C. Patel College of Allopathic Medicine, Fort Lauderdale, USA

**Keywords:** peripheral edema, heart failure, lower limb edema, compression stockings, edema management

## Abstract

Peripheral edema is a prevalent condition affecting patients dealing with an assortment of health conditions, such as congestive heart failure (CHF), liver disease, venous insufficiency, and postoperative surgical complications. Edema can present in a variety of ways, ranging from mild localized symptoms to severely debilitating forms that impact patients’ daily lives. Despite the vast number of publications addressing the underlying causes of peripheral edema, there seems to be an absence of literature that presents the effectiveness and compliance of current management techniques. This paper aims to condense the current literature on the effectiveness and compliance of current edema management approaches across various common etiologies, with the intention of identifying alternative therapies that could enhance the quality of care for patients with chronic lower extremity edema. Several promising new therapies such as exogenous calf muscle stimulation, leg raise exercises, high-dose albumin injections, and device-based negative pressure lymph drainage (NPLD), deviate from the current established standard of care. This scoping review revealed diverse treatment methods tailored to the specific underlying etiology of edema. The use of diuretics and vasodilators has shown benefits in treating CHF-induced edema but failed to alleviate and prevent the recurrence of edema in hospitalized and recently discharged patients. Albumin injections have emerged as a potential alternative treatment for edema due to liver disease, addressing hypoalbuminemia symptoms caused by liver failure. Patients with vascular causes of edema are efficaciously treated conservatively with compression stockings, although patient adherence remains a hurdle. For postoperative edema, device-based NPLD appears promising, with potential benefits over elastic bandage wraps and kinesiology taping.

## Introduction and background

Peripheral edema is a complex condition that affects patients dealing with an assortment of diseases and thus its underlying pathology varies considerably. Some of the most common causes include congestive heart failure (CHF), liver disease, vascular conditions such as chronic venous insufficiency, and postoperative surgical complications [1]. Edema is balanced by two counteractive forces, hydrostatic pressure and oncotic pressure. When this delicate homeostatic mechanism becomes dysregulated, either by an increase in hydrostatic pressure or a decrease in oncotic pressure, it can lead to increased capillary permeability and fluid buildup in the interstitial space, known as edema. Hydrostatic pressure is the force that allows fluid to be filtered out of the capillaries and into the interstitial space. This pressure can rise from fluid accumulation or any circulatory obstruction that promotes fluid pooling and congestion [1]. In contrast, oncotic pressure is the force that works to ensure fluid remains in the capillaries and is maintained by intravascular proteins like albumin, which draw fluid back into the capillaries [1]. Edema can present in a variety of ways, from benign localized symptoms to the development of a severely debilitating and disfiguring condition that plagues patients daily. 

The presence of CHF significantly increases the risk of peripheral edema via venous fluid overload as the heart becomes incapable of pumping blood effectively throughout the circulatory system, ultimately leading to an increase in hydrostatic pressure. Right-sided heart failure can consequently develop when left-sided heart failure and pulmonary congestion obstruct the outflow of blood from the right ventricle. Right-sided heart failure results in venous congestion, blood stasis, and swelling below the level of the heart. Left-sided heart failure can develop when chronic hypertension or a myocardial infarction results in a decrease in cardiac output from the left ventricle [2]. Reduced cardiac output initiates a cascade of counter-regulatory measures to compensate for the reduced blood flow circulating throughout the body, including activation of the renin-angiotensin system, increased antidiuretic hormone secretion, and enhanced retention of sodium and water [2]. These counter-regulatory measures contribute to edema formation through their fluid-retaining properties. Lower extremity edema is so often associated with CHF that ankle edema is a minor criterion for the diagnosis of heart failure, according to the well-established Framingham criterion [3]. Current treatment guidelines for CHF-related peripheral edema are centered around pharmacotherapy, mostly diuretics [2]. Diuretics are determined to be advantageous in reducing fluid retention in the systemic circulation, whereas vasodilators play a greater role in decreasing venous pressure in the pulmonary circulation [4].

The liver is another key organ system that can be responsible for peripheral edema. Liver failure contributes to increased peripheral edema through multiple mechanisms of action. One way in which hepatic disease can cause bilateral edema is through the impairment of the liver’s ability to synthesize albumin. This, in turn, will decrease the oncotic capillary pressure, ultimately allowing more fluid to leave the blood vessels and enter the extracellular space. Liver failure is also a contributing factor to peripheral edema due to increased hydrostatic pressure in systemic capillaries resulting from portal vein congestion. This congestion develops in the advanced stages of liver disease when blood flow through the liver is impeded, leading to increased venous pressures, fluid buildup, and swelling. The aldosterone antagonist spironolactone is currently being used as a primary treatment either independently or in combination with a loop diuretic for edema stemming from liver disease [5]. Spironolactone and loop diuretics both function in reducing fluid retention to lower hydrostatic pressure and alleviate edema.

Edema due to vascular problems can arise from chronic venous insufficiency, which is a failure of the valves in the veins of the legs to close properly, impeding the upward flow of blood towards the heart [1]. This results in the gravitational pull of fluids into the legs, leading to blood pooling and heightened hydrostatic pressure within the veins. Over time the accumulated fluids permeate the surrounding tissue, causing swelling and local ischemia that may lead to ulcers [1]. Prolonged standing, sitting, or increased sodium intake may potentially exacerbate this problem. Another vascular factor contributing to lower extremity edema may be due to deep vein thrombosis (DVT), which is a clot that forms in the deep veins of the lower extremity [6]. DVTs can obstruct affected veins, causing stasis and the pooling of blood, or may even redirect blood to alternative veins resulting in increased venous pressure, swelling, and pain [6]. The approach to managing edema caused by vascular issues is guided by the predisposing condition that leads to the edema. Chronic venous insufficiency is commonly treated long-term with mechanical therapies such as compression stockings as a way to help redirect fluid back towards the heart against gravity. In cases where chronic venous insufficiency can be attributed to DVT, newer anticoagulants (rivaroxaban or apixaban) or low molecular weight heparin are recommended to reduce the risk of clot formation [1]. 

Following a traumatic event, such as orthopedic surgery, an inflammatory process is triggered leading to the release of key inflammatory mediators that increase capillary permeability. As a result, more fluid concentrates in connective tissue [7]. This excess fluid overwhelms the lymphatic system, which is primarily responsible for draining and returning it to circulation [7]. Over time, the compromised drainage process of the lymphatic system struggles to manage the excess fluid, leading to increased hydrostatic pressures and eventual edema. Standard conservative treatments for lower limb postoperative edema stemming from orthopedic surgeries are typically treated with positive pressure methods such as manual lymphatic drainage, compression wraps, or compression devices as a way to force excess fluid out of the extremities [7]. Furthermore, physical therapy aimed at improving limb range of motion and alleviating edema is employed in particular instances of edema stemming from intramedullary nailing for the treatment of femoral shaft fractures [8].

The diverse causes and locations of edema among patients have created a treatment barrier as there is no one-size-fits-all approach. For instance, someone who is suffering from peripheral edema caused by diabetes will be treated differently than someone who has edema stemming from underlying cardiac issues. This variability in treatment can create a challenge for physicians to find not only the most effective treatment on a case-by-case basis but also one that will ensure patient compliance. Prolonged edema comes with a range of complications, each requiring distinct techniques, including addressing the range of motion of various limbs in many cases along with overall quality of life. Patient compliance with treatment is an issue that is exacerbated by chronic conditions. The chronic nature of the condition makes it more difficult for patients to consistently adhere to their treatment regimens. Therefore, this scoping review aims to answer if and when particular management techniques may prove more efficacious than the current standard of care in certain chronic conditions, while simultaneously catering to the diverse and unique preferences of the patient. This information should empower clinicians to make well-informed clinical decisions for their patients' betterment. 

Methodology

The study aimed to review the effectiveness and compliance of current lower extremity edema management techniques for patients suffering from chronic edema due to CHF, liver disease, vascular insufficiency, or postoperative complications using the Preferred Reporting Items for Systematic Reviews and Meta-Analyses (PRISMA) guidelines published by the Joanna Briggs Institute (JBI) (www.jbi.global). 

Population, Concept, Context (PCC) Criteria 

The research question was formulated using the population, concept, context (PCC) protocol, where the population (P) consisted of males and females aged 18 or older suffering from edema due to chronic pathologies, such as CHF, liver disease, vascular insufficiency, and postoperative complications; the concept (C) being alternative treatments to the current standard of care for edema; and the context (C) being studies conducted within developed countries.

Inclusion/Exclusion Criteria 

To refine the investigation, inclusion/exclusion criteria were created to help with the identification of relevant journal articles. Inclusion criteria stipulated that the patient population studied be males and females aged 18 years or older suffering from chronic edema due to CHF, liver disease, vascular insufficiency, or postoperative complications. Studies were only included if they were conducted from January 1, 1990, to October 1, 2022, and were published in English. Case studies and narrative review papers were excluded from the analysis.

Database Searches 

To find articles specifically tailored to the study’s inclusion/exclusion criteria, the databases PubMed, Elsevier, and EBSCO were searched. All relevant journals were subsequently imported into Rayyan (www.Rayyan.ai) so that each of the researchers could signal whether an article was to be approved or rejected in a blinded fashion. The search process was not supplemented with hand-searching for journals or scanning of reference lists. The process was documented using the PRISMA flow diagram [9]. Searches were carried out on August 6, 2022. 

Databases were searched using the following keywords and Boolean operators: “edema” AND “treatments”, “vascular edema”, “lower extremity” AND “edema”, “post surgery edema” AND “lower extremity”, “cardiovascular failure” AND “edema”, “liver failure” AND “edema”, “heart failure” AND “edema”, “vascular complications” AND “edema”, “deep vein thrombosis” AND “efficacy”, “venous stasis” AND “treatment” AND “efficacy”, and “lymphedema” AND “treatment”. 

Data Extraction 

Data was extracted using the template provided by JBI [10]. Ten investigators worked in groups of two to extract data from articles based on their assigned inclusion criteria. The data was extracted onto a premade template on Microsoft Excel (Microsoft Corporation, Redmond, USA). 

Quality Control 

Quality control for included articles was performed using the JBI criteria relevant to their respective study type [10]. A total of 15 studies were evaluated using the JBI critical appraisal checklist for randomized controlled trials [10]. Additionally, two studies were appraised using the critical appraisal checklist for systematic reviews, while two others were evaluated using the critical appraisal checklist for cohort studies [11]. One study was assessed using the critical appraisal checklist for textual evidence: expert opinion, and one other was evaluated using the critical appraisal checklist for analytical cross-sectional studies [12]. Studies were added to the approved list if they were deemed as a low risk for bias, which was defined as having a score of ≥70%. All studies <70% were deemed as a high risk for bias.

## Review

**Figure 1:**
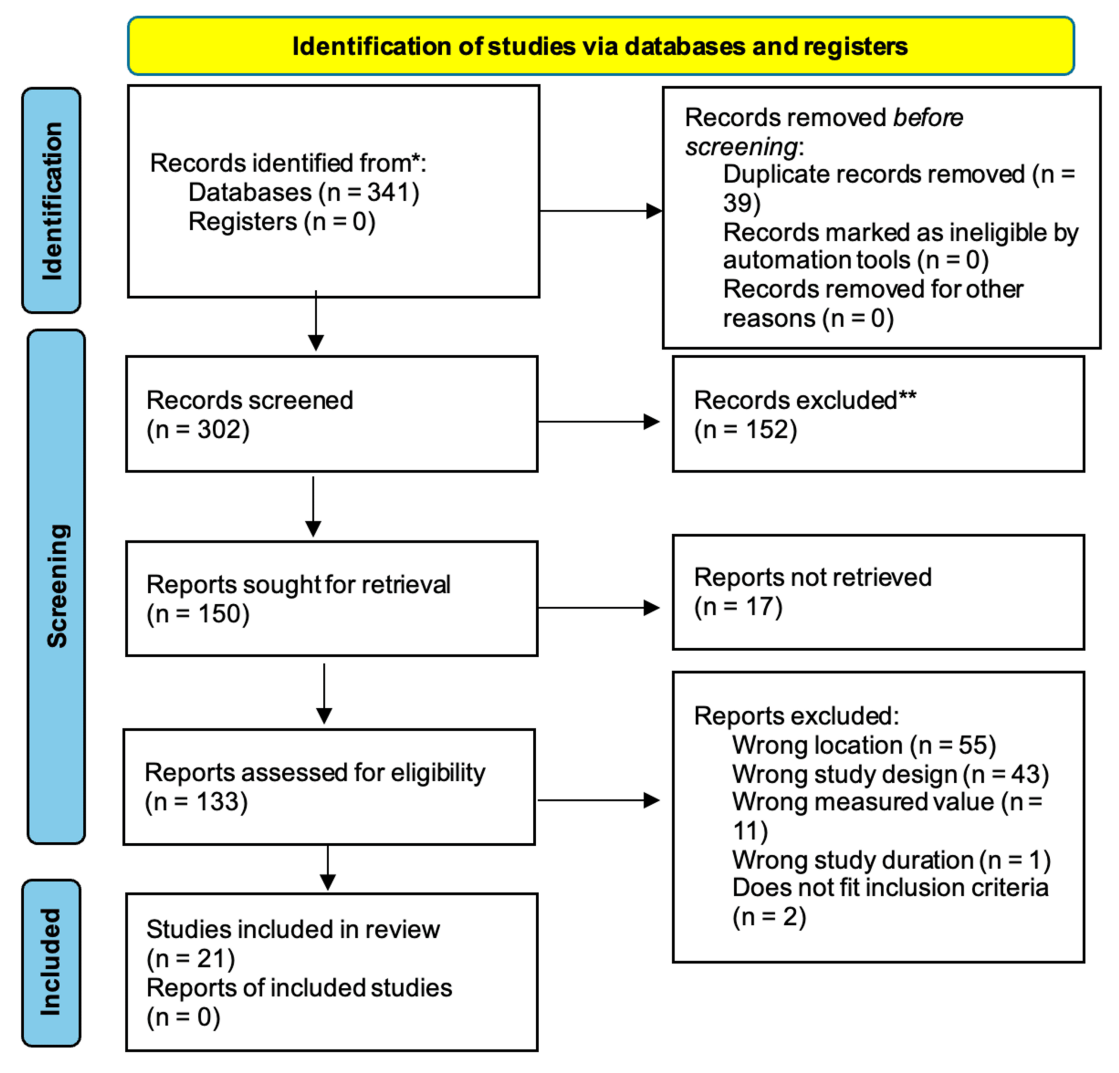
PRISMA flow diagram PRISMA: Preferred Reporting Items for Systematic Reviews and Meta-Analyses

Out of the 302 records screened, the application of the final inclusion and exclusion criteria yielded 21 articles (Figure 1). Studies were performed in the United States (13), Japan (1), Italy (3), Switzerland (2), Poland (1), and Spain (1). Once the scoring criteria were completed, all 21 articles picked were categorized based on study type and organ system. Of these 21 articles, four focused on edema due to CHF, three on edema due to liver failure, eight on edema due to vascular insufficiency, and six on postoperative edema. Table 1 summarizes the studies included in this review. 

**Table 1:** Summary of included studies CHF: Congestive heart failure; IPC: Intermittent pneumatic compression; QOL: Quality of life

Etiology Focus	Study	Study Aim	Treatments Discussed	Current Standard of Care?	Key Findings
CHF	Clark and Cleland [4]	Describe the pathophysiology of edema due to CHF and discuss how it can guide treatment.	Diuretics Vasodilators	Yes	(1) Vasodilators can assist in lowering the venous pressure in the pulmonary circulation. (2) Diuretics can be used to reduce the fluid in the systemic circulation, thereby decreasing venous pooling in the extremities.
CHF	Lala, McNulty, and Mentz et al. [13]	How the clinical components of congestion affect outcomes of patients treated for acute decompensated heart failure after hospital discharge.	Diuretics	Yes	(1) Current standards of care are inadequate in treating edema in hospitalized and recently discharged patients. (2) Higher orthodema scores are associated with increased adverse events post-discharge.
CHF	Metra, Bugatti, and Bettari et al. [14]	Explore new treatments for relieving congestion in heart failure, including overcoming furosemide resistance and kidney protection.	Furosemide (loop diuretic)	Yes	(1) Furosemide failed to relieve symptoms in up to 50% of patients due to drug resistance and adverse side effects.
CHF	Pierce and McLeod [15]	Feasibility of using calf muscle pump stimulation to reduce lower extremity edema.	Exogenous calf muscle stimulation	No	(1) Exogenous calf muscle stimulation reduced the lean mass of the legs by 0.5 kilograms. (2) Increased use of the stimulator was associated with water loss, but the results were not drastic.
CHF	Lee, Lennie, and Dunbar et al. [16]	To find if monitoring lower extremity edema could be a predictor of cardiac event-free survival in patients with heart failure.	Frequent monitoring of lower limb edema	N/a	(1) Patients non-compliant with edema monitoring had a 1.9 times higher risk of cardiac event. (2) Frequent monitoring of lower extremity edema improves cardiac outcomes.
Liver disease	Sakaida, Terai, and Kurosaki et al. [5]	Evaluate the effectiveness and safety of tolvaptan in liver cirrhosis patients with hepatic edema.	Tolvaptan	No	(1) Following tolvaptan treatment, body weight decreased from baseline by 2.6-2.7 kilograms on day seven and by 3.8-4.1 kilograms on day 14. (2) Tolvaptan is effective and well-tolerated in liver cirrhosis patients with hepatic edema.
Liver disease	Fernández, Clària, and Amorós et al. [17]	To investigate the effects of therapeutic albumin infusions on cardiocirculatory dysfunction, hypoalbuminemia, and portal hypertension in patients with decompensated cirrhosis.	High-dose albumin infusions	No	(1) Albumin infusions decreased cardiocirculatory dysfunction and systemic inflammation.
Liver disease and postoperative	Mathews, James, and Anderson et al. [18]	Researchers aimed to investigate the effectiveness of compression wraps in managing edema in both pre- and post-liver transplantation.	Compression wraps	Yes for postoperative edema, but not for edema caused by liver disease.	(1) After a single eight-hour treatment with compression wraps, the average circumference decreased by 2.6 centimeters at the ankle and 3.1 centimeters at midcalf. (2) In contrast, the average circumference only decreased by 0.4 centimeters at the ankle and by one centimeter at midcalf in unwrapped legs.
Venous insufficiency	Ciocon, Daisy Ciocon, and Diana Galindo et al. [19]	To see if patients with leg edema who perform raised-leg exercise (20 minutes three times a day) show improvement in leg edema measurements compared to those who do not.	Raised-leg exercises	No	(1) A significant reduction in leg edema resulted from performing raised-leg exercises for 20 minutes three times a day. (2) Same results were not seen in patients who had edema due to drug use, heart failure, or other etiologies.
Venous insufficiency	Wu, Crews, and Andersen et al. [20]	Efficacy of mild compression socks on lower extremity edema in diabetic patients without negatively impacting their vascular supply.	Compression stockings	Yes	(1) Compression stockings significantly reduced edema as measured by calf and ankle circumference. (2) Adverse events such as decreased lower extremity circulation were not observed.
Venous insufficiency	Tessari, Tisato, and Rimondi et al. [21]	To see the effects of IPC on patients with leg edema and reduced mobility.	IPC	Subtype of the current standard of care	(1) IPC resulted in significant improvement in leg edema and ankle range of motion, QOL, and modulation of plasma inflammatory markers.
Venous insufficiency	Raju, Hollis, and Neglen et al. [22]	The use, efficacy, and compliance of compressive stockings in patients with chronic venous disease.	Compressive stockings	Yes	(1) Symptoms persisted in 37% of patients despite compliance. (2) Non-compliance stemmed from stockings not being prescribed by the primary physician (25%), lack of improvement (14%), cutting off circulation (13%), and being too hot to wear (8%). (3) Compressive stockings cannot be utilized in about a quarter of patients due to limb condition or general health.
Venous insufficiency	Schulman, Granqvist, and Holmstrom et al. [23]	To reach a consensus on the optimal duration of oral anticoagulant therapy following a second venous thromboembolism.	Indefinite anticoagulation therapy (warfarin sodium or dicumarol)	No	(1) Indefinite prophylactic anticoagulation following a second venous thromboembolism was associated with a significantly lower recurrence rate during four years of follow-up (compared to six months of anticoagulation).
Venous insufficiency	Alsheekh, Hingorani, and Ferm et al. [24]	To identify clinical factors including race and ethnicity related to persistent leg swelling after treatment with iliac vein stenting and thermal ablation.	Iliac stenting and venous ablation	No	(1) Clinical factors like race are not significant in determining swelling response to an iliac stent and endovenous ablation procedures. (2) Higher degree of iliac vein stenosis is associated with improved resolution of swelling.
Venous insufficiency	Brathwaite, Minton, and Benarroch-Gampel et al. [25]	The study aims to retrospectively review the early outcomes for the largest U.S. series of patients who have undergone popliteal vein external banding.	Popliteal vein external banding	No	(1) 75% of patients with an active ulcer healed completely. (2) 91.6% of patients reported clinical improvement with a reduction in edema, pain, or size of ulcer.
Venous insufficiency	Aurshina, Zhang, and Zhuo et al. [26]	The study aims to investigate the safety and efficacy of venous ablation in octogenarians.	Venous ablation	No	(1) Venous ablation resulted in less severe ultrasound findings. (2) Venous ablation is safe and effective for all age groups.
Postoperative	Dresing, Fischer, and Lehmann et al. [7]	Utilization of a negative pressure device (LymphaTouch) for post-surgery recovery and measurement of its effectiveness in reducing postoperative swelling.	LymphaTouch	No	(1) LymphaTouch allowed interstitial fluid to flow through soft tissues and drain into local lymphatic capillary pores. (2) An 11.6% measurable decrease in swelling of the lower extremity was reported after four treatments.
Postoperative	Lanier, Johnson, and Tapia et al. [8]	Compare the effects of kinesiology taping on lower limb edema following intramedullary nailing for femoral shaft fracture.	Kinesiology tape	No	(1) Kinesiology tape did not significantly reduce the volume of the lower limb, reduce pain, or improve postoperative mobility.
Postoperative	Scaglioni, Meroni, and Fritsche [27]	To describe the treatment of lymphaticovenous anastomosis for lymphedema with superficial and deep lymphatic vessels.	Lymphaticovenous anastomosis	No	(1) Patients had a reduction in swelling and relief from the sensation of heaviness. (2) Postoperative courses proceeded without complications. (3) No recurrence of the condition at nine months follow-up, even with complete removal of compressive therapy.
Postoperative	Maccarone, Venturini, and Menegatti et al. [28]	Assess the outcome of aquatic exercise on limb motor function, QOL, limb volume, and pain in patients with primary or secondary lymphedema.	Aquatic exercises	No	(1) Aquatic exercise improved range of motion, pain sensation, QOL, and limb strength. (2) QOL appeared to diminish over time in control groups. (3.) Most studies included reported decreased limb volumes, while a few observed no benefits.
Postoperative	Zaleska and Olszewski [29]	To measure additional parameters useful for the understanding of tissue and fluid events and for the approval of the Linforoll device for general practice.	Linforoll device	Subtype of the current standard of care	(1) Hydromechanic parameters of the skin remained normal under high massaging pressures, indicating a lack of destructive changes. (2) Subcutaneously accumulated edema fluid can be moved proximally under pressures of 80-120 mmHg.

 *Congestive Heart Failure (CHF)*

Three studies pertained to the use of diuretics [4,13,14]. Two of the three studies highlighted the effectiveness of diuretics in the management of peripheral edema caused by CHF [4,13]. Research involving patients enrolled in the Diuretic Optimization Strategy Evaluation found that diuretics decreased orthodema score, which is a score that reflects the severity of congestion throughout the body and is associated with a lower risk for adverse events following discharge [13]. One study concluded that the loop diuretic furosemide was not an ideal treatment method for managing venous congestion associated with CHF, as it failed to relieve symptoms in up to 50% of participants due to high drug resistance and adverse effects [14].

The remaining three studies evaluated alternative clinical approaches to managing edema from CHF. The use of exogenous calf muscle stimulation decreased lower extremity fluid retention in elderly patients with chronic CHF, but the results were not drastic [15]. Performing raised-leg exercises for 20 minutes three times a day on elderly patients with leg edema had no improvement in edema caused by CHF [19]. Frequent monitoring of lower extremity edema in patients with CHF improved cardiac outcomes. Patients who did not adhere to this monitoring were 1.9 times more likely to experience adverse cardiac events such as death, hospitalization, or visits to the emergency department for cardiac reasons [16].

*Liver Disease* 

The scoping review’s inclusion criteria rendered three liver-centric studies that investigated edema in patients with hepatic cirrhosis. A 14-day tolvaptan treatment, which is a vasopressin antagonist and antidiuretic hormone inhibitor, resulted in a marked reduction in body weight (an indicator of hepatic edema), ascites volume, lower limb edema, and pleural effusion in patients with liver failure [5]. Improving patient conditions with individuals suffering from decompensated cirrhosis was also made possible by administering chronic, high-albumin dose (HAlbD) treatments [17]. HAlbD treatments also resulted in marked reductions of pro-inflammatory markers - interleukin-6 and cytokines. Combining albumin with antibiotics showed an even greater decrease in the level of systemic cytokines compared to those that were given antibiotics alone [17]. Elevation of cardiac index, systolic volume, and left ventricular stroke work index also signaled an improvement in left ventricular function stemming from HAlbD treatment [17]. The final study demonstrated that the application of elastic bandage wraps for eight hours showed significantly less edema formation and pain in patients’ legs prior to undergoing liver transplantation [18]. 

Venous Insufficiency

Five studies investigated conservative approaches to edema stemming from venous insufficiency. While some of the studies focused on the effectiveness of compression stockings, the mainstay treatment for vascular edema, others explored conservative alternative therapies [19-22]. Several studies investigated leg-raise exercises and intermittent pneumatic compression (IPC) calf muscle pumps [16,21]. Studies assessing compression socks and IPC calf muscle pumps consistently reported significant decreases in both calf diameter and leg swelling within their respective study periods [20-22]. A significant decrease in lower extremity edema among patients with venous stasis etiologies resulted from performing leg-raise exercises three times a day for 20 minutes when compared to a control group [19]. One study investigated the optimal duration of oral anticoagulant prophylaxis following a second DVT, revealing that indefinite prophylaxis led to a significant decrease in recurrence over a four-year follow-up compared to a six-month prophylaxis period, thereby reducing the recurrence of lower extremity edema from DVT [23].

Three studies investigated invasive methods like venous ablation, iliac vein stenting, and popliteal external banding for edema due to venous insufficiency [24-26]. Venous ablation in individuals in their 80s revealed milder ultrasound findings compared to younger patients, indicating that age should not be a deterrent for considering venous ablation as a viable treatment option [25]. Popliteal external banding for lower extremity edema resulted in significant improvements in ulcer pain and healing (75%) and edema (91.6%) [25]. A study investigating persistent leg swelling following treatment with iliac vein stenting and thermal ablation found no significant difference in swelling between these two procedures [24]. 

Postoperative 

The scoping review’s inclusion criteria encompassed six articles addressing edema in postoperative patients. Only one study investigated invasive treatments for postoperative edema. A decrease in lower limb swelling alongside a relief from the sensation of heaviness was found with trials of the invasive surgical procedure, lymphaticovenous anastomosis (LVA) [27]. Five studies investigated noninvasive approaches [7,8,18,28,29]. One study demonstrated the Linforoll device’s effectiveness in reducing lower limb edema by moving subcutaneous fluids in edematous conditions under pressures of 80-120 mmHg without causing skin damage [29]. Linforoll is a device consisting of a handpiece, a roller, and a sensor linked to a computer for visualizing the pressure curve of the applied force. A perioperative and posttraumatic approach to limit edema with a decongestive device based on negative pressures led to an 11.6% decrease in lower extremity edema after four treatments [7]. Patients in this study also experienced better mobility and decreased levels of pain [7]. Treatment of lymphedema with aquatic exercises showed improvements in range of motion, calf circumference, pain reduction, and overall quality of life compared to the control group [28]. Another study discussed the use of elastic bandage wraps for individuals who recently underwent liver transplantation [18]. The study found that applying elastic bandage wraps to one leg resulted in reduced pain and significantly less edema in the treated leg compared to the non-wrapped leg [18]. A retrospective study conducted on the use of kinesiology taping following intramedullary nailing for femoral shaft fractures demonstrated improved active knee extension as a result of quadriceps force enhancement following the application of kinesiology tape [8]. While the study did not find a significant reduction in pain or edema using this treatment method, the enhanced ability to apply force with the quadriceps muscle was significant [8].

Discussion

**Figure 2:**
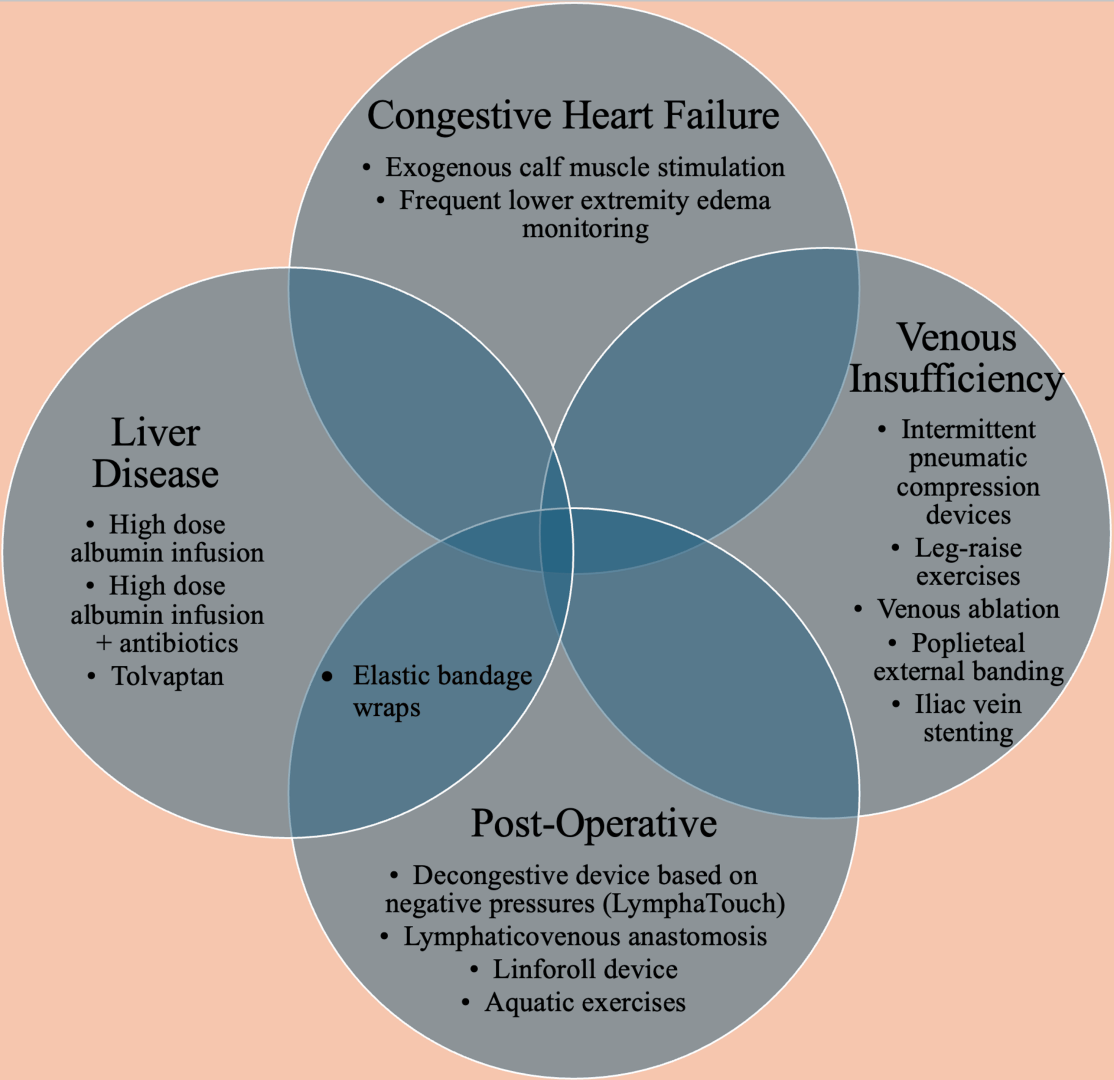
Alternative therapies for edema management classified by the underlying cause Image Credits: Blake Smith

The optimal treatment regimen for managing edema is complex and varies depending on the underlying cause, how well the patient tolerates and adheres to the treatment regimen, and the personal preferences of the patient, among other factors. This review described therapies for edema that may be considered as an alternative treatment to the typical standard of care. These alternative treatments may be more suitable for particular subsets of patients with edema, depending on both the underlying cause of the condition and the unique conditions and preferences of the patient. Figure 2 illustrates these alternative therapies for treating edema arising from different etiologies, diverging from the usual standard of care. These alternative therapies could currently be useful as an adjunctive therapy alongside current standards of care, especially in individuals non-compliant or resistant to particular therapies.

Limitations of Current Standards of Care

Pharmacological interventions like diuretics and vasodilators used to treat edema from CHF, align with current standards that prioritize these treatments for CHF-related fluid management. Despite their widespread use, this review showed a gap in efficacy for managing edema during hospitalization and in preventing its recurrence after discharge [13]. In the context of liver disease, where edema formation is predominantly due to decreased oncotic pressure from reduced albumin synthesis, the standard treatment involving spironolactone and loop diuretics was often effective. However, in severe cases of edema due to liver disease and hypoalbuminemia, traditional diuretics may not be as effective as they are unable to restore serum albumin levels. Some patients with liver disease have also been reported to exhibit signs of diuretic resistance, ultimately preventing them from responding adequately to the current standard of care [5]. This review further supported the use of compression stockings as a primary treatment for edema caused by venous insufficiency. Nonetheless, some patients experienced difficulty tolerating this type of therapy due to discomfort, excessive warmth, or compromised circulation caused by the stockings themselves, or due to the poor condition of the limb requiring treatment [22]. Currently, the available evidence regarding the efficacy of positive pressure techniques and devices for postoperative edema is largely limited [7].

Alternative Treatments and Physician Recommendations

Physicians should be cognizant that diuretics may not always be the ideal treatment modality for edema stemming from congestive CHF when factoring in the unique circumstances and preferences of the patient. Loop diuretics are notorious for their short half-life, making the administration of several daily doses a necessity for them to be effective [1]. This could become problematic in patients who may be more prone to forgetting their daily medication administration. While diuretics have proven their worth as the most effective treatment in managing edema from CHF, some patients may experience adverse side effects such as electrolyte abnormalities, hyperuricemia, and renal failure [15]. In these situations, physicians may be resourceful in remembering that exogenous calf muscle stimulation has value in reducing lean mass in edematous legs through the prevention and reversal of fluid pooling in the lower limbs [15]. Exogenous calf muscle stimulation, although not as effective as loop diuretics, could serve as an adjunct therapy, especially for patients who are non-compliant or who are experiencing adverse effects from loop diuretics. Its favorable safety profile and non-invasive nature may permit its use as an adjunct therapy in those on optimal medical therapy. As frequent monitoring of lower extremity edema was determined to improve patient outcomes, it can be safely argued that physicians placing a greater emphasis on follow-up care regarding lower extremity edema in these patients would be beneficial to decrease the risk of adverse cardiac events [16].

For individuals suffering from edema caused by liver disease that hasn’t responded well to traditional treatment modalities, there are other options to explore. Physicians may potentially consider tolvaptan, HAlbD treatments, or compression wraps as an adjunct therapy to conventional approaches. This could be particularly relevant to patients who are unable to take or are resistant to loop diuretics. Tolvaptan can theoretically reduce fluid overload and hydrostatic pressures through its inhibition of antidiuretic hormone. Physicians should particularly emphasize HAlbD treatments as an alternative therapy in patients with severe hypoalbuminemia, as loop diuretics do not restore serum albumin levels as a way to increase oncotic pressure and reduce edema. Although HAlbD infusions show promising results, administering albumin too quickly can result in up to a fourfold increase in fluid retention and edema [30]. 

Although compression stockings are the primary treatment modality for edema caused by venous insufficiency, patient adherence remains an issue. Physicians may consider alternative conservative therapies such as intermittent pneumatic compression calf muscle pumps and raised leg exercises as adjunct therapy to treat edema in patients who are unwilling or unable to tolerate compression stockings due to discomfort, excessive warmth, or restricted circulation. If traditional conservative approaches prove ineffective, physicians may consider innovative invasive treatments like venous ablation, popliteal vein external banding, or stenting of the iliac vein. Venous ablation offers an invasive approach to treating lower extremity edema using laser or radiofrequency techniques; however, nerve injury, phlebitis, and hematomas were reported as potential complications of the procedure [26]. External banding of the popliteal vein is an invasive technique that involves placing a vascular graft around the outside of the popliteal vein as a way to combat venous reflux and fluid pooling in the lower extremities [31]. While this approach may be considered one of the easier procedures compared to other deep venous reconstructive options, some patients experience postoperative wound complications [25]. Stenting of the iliac vein is an invasive approach that consists of inserting a stent into the iliac vein to maintain its patency and proper fluid circulation, but there is a risk of stent restenosis [32].

Aquatic exercises have demonstrated effectiveness as an alternative therapy for reducing lower limb volumes in edematous patients. Physicians should consider adding this adjunct therapy to treatment regimens for patients inadequately responding to conventional positive pressure compression therapies. Investigating innovative modalities for postoperative edema, device-based negative pressure lymph drainage (NPLD) showed the most potential. Device-based NPLD demonstrated a marked decrease in edema and pain, offering a promising conservative alternative to conventional methods like compression therapy. Furthermore, the addition of kinesiology taping as an adjunct therapy to the treatment of lower limb postoperative edema may result in patients having increased strength with hip flexion and knee extension. Physicians may contemplate innovative invasive procedures like LVA in cases where conservative approaches continue to fail.

Review Limitations

The scope of this review was restricted by its small selection of databases, its use of only a few, specific search terms, and its focus on publications written only in English and that were peer-reviewed. Limitations in the scope of this review potentially omitted valuable data from international studies or alternative medical practices not commonly included in Western literature. This could skew the comprehensiveness of our analysis and the applicability of our recommendations across different global contexts. The incorporation of additional synonymous search terms could have possibly produced a greater number of studies than our review did. Moreover, the primary studies reviewed often had methodological limitations such as small sample sizes and short-term follow-ups which may impede the reliability of their outcomes. The review process encountered challenges regarding postoperative edema due to the variability of surgical procedures capable of producing it. This variability made it difficult to compare treatment techniques, as not all causes were equivalent, resulting in patients having various degrees of edema. 

Research on exogenous calf muscle stimulation for edema due to CHF was notably limited by a very small sample size and treatment duration. A limitation of the study on HAlbD treatments for edema stemming from liver disease is that it measured centrally located ascites as opposed to directly measuring lower extremity edema [17]. This implicates a further need for research in the field of albumin infusions and its effects directly on lower extremity edema. Methods of decreasing lower limb edema via elastic bandage wraps for patients with liver disease were also explored, and despite a lack of trials on long-term usage, provided some insight into lowering edema [18]. Long-term investigation of these methods is required for confirmatory results. The study on popliteal vein external banding as a treatment for edema resulting from venous insufficiency was limited by a small sample size. Research regarding kinesiology tape in treating postoperative edema was limited by low test-retest reliability of lower extremity circumferential measurements in addition to involving a small sample size [8]. 

## Conclusions

The literature contains evidence that a number of alternative treatments may provide some benefit to patients experiencing edema - either as stand-alone treatments or as adjunctive treatments to the current standard of care. CHF-induced edema treatments ranged from the utilization of loop diuretics to exogenous calf muscle stimulation. Diuretics, with the exception of furosemide, proved most beneficial in the treatment of those with edema stemming from CHF. Continual monitoring of lower limb edema in patients with CHF results in improved cardiac outcomes. Researchers have shown several different treatment modalities to be successful in managing edema in patients undergoing liver failure. Treatment plans can be individualized for patients whose symptoms have failed to improve under more traditional treatment modalities. These alternative treatments, such as tolvaptan and HAlbD administration, can be used in liver failure patients who are contraindicated or have shown negligible improvement with loop diuretics. There are both nonsurgical and surgical solutions available to alleviate edema in patients with chronic venous insufficiency; however, the effectiveness of such treatments is variable and dependent upon patient tolerability and preference. For postoperative edema, device-based NPLD appears promising as an alternative conservative treatment to the current standard of care, with potential benefits over elastic bandage wraps and kinesiology taping. Further research with larger sample sizes and longer durations, while simultaneously considering confounding variables such as age and sex, is required to obtain a more reliable scope on the efficacy of the many treatments discussed. 
